# Estimating under-observation and the full size of the 2016 Zika epidemic in Rio de Janeiro

**DOI:** 10.1371/journal.pone.0205001

**Published:** 2018-10-12

**Authors:** Marcio M. Bastos, Flávio Codeço Coelho

**Affiliations:** Escola de Matemática Aplicada, Fundação Getúlio Vargas, Rio de Janeiro, RJ, Brazil; Columbia University, UNITED STATES

## Abstract

The 2015-16 Zika epidemic spread quickly from north to south in Brazil. Two striking features were the much higher incidence in young adult women due to sexual transmission, and the serious congenital malformations and miscarriages associated to Zika infection in pregnant women. In this paper we use case reporting data along with live-birth records to reconstruct the full size of the epidemic through a Bayesian probabilistic graph model representing the Zika transmission probabilities of observation (case reporting) and of birth loss (through miscarriage or abortion). We find that the probability of observing (reporting) a Zika case is different between men and women and ranges between 10 to 13%. From these estimates we reconstruct the full size of the Zika epidemic in Rio de Janeiro in 2015-16.

## Introduction

In the summer of 2015-16, the city of Rio de Janeiro suffered from a epidemic of Zika which had been introduced months earlier in the northeast of the country. The quick spread of Zika and the particular way in which it attacked the female population [1] led to serious public health consequences. Rio de Janeiro had one of the country’s largest incidences of microcephaly associated with Zika. Another important impact was the marked number of lost pregnancies associated with Zika [2].

Despite its serious implications, the clinical diagnosis of Zika in Rio was not easy because of the combination of mild symptoms and the difficulty of distinguishing it from the simultaneous outbreaks of Dengue and Chikungunya taking place in the city.

Nevertheless, the speed with which the epidemic covered the entire country seems to point to a high transmissibility and a low observation rate [3, 4]. Here we define observation as a clinically diagnosed patient. Due the high state of alert of the health system, a fairly high notification rate (given a clinical diagnosis) was maintained throughout the epidemic.

Estimating the parameters that govern its transmission, specially the *basic reproduction number* ($R_{0}$) [5], is important to comprehend its dynamics, because it gives a metric to compare the spread of the disease in different areas and to help automatically detect patterns, leading to more accurate forecasts and better prevention, therapy and control [6].

The Zika outbreak has already been studied in other regions with different probabilistic approaches such as the study of the transmission dynamics during the epidemic in 2013-2014 in French Polynesia [7] and the estimation of the Reproduction Number and the role of the sexual transmission of the epidemic in 2015 in Barranquilla, Colombia [8].

As one of our goals in this paper is to estimate the under-reporting rate, we use case reporting data which also give us reliable estimates about the relative incidences by age and gender. The complete birth record in Rio de Janeiro, including the drop in births associated to female cases of Zika during the 2016 epidemic [2], helps us estimate the probability of observing a case of Zika, and consequently the full size of the epidemic in the city of Rio de Janeiro.

This paper makes two key contributions to the understanding of Zika: firstly, it provides a realistic estimate of the real magnitude of the Zika epidemic in Rio de Janeiro in the year 2016 and secondly it measures the drop in births caused by Zika [2] through a non-iterative methodology for the estimation of parameters of an SIR transmission model using Bayesian inference.

## Methods

In order to estimate the global epidemiological parameters for Zika from the available data, we approximated the transmission dynamics by an SIR model. In this model, we assumed that the total population in the city is constant in the study period (*N* = *S*(*t*) + *I*(*t*) + *R*(*t*), ∀*t*) and is divided into the susceptible, infectious and removed classes. The dynamics between these classes are governed by the Eqs (1), (2) and (2). This approximation of vector-borne transmission by an SIR model has been applied successfully before [9] and is justifiable due to the lack of specific data on the vector.

$$\frac{dS}{dt}=\frac{-\beta IS}{N}$$ (1)

$$\frac{dI}{dt}=\frac{\beta IS}{N}-\gamma I$$ (2)

$$\frac{dR}{dt}=\gamma I$$ (3)

Although Zika is transmitted *via* both mosquitoes and sexual contact [1] the total transmissibility is represented by a single parameter *β* in the model used here. As the time period considered is short, a single recovery rate *γ* is also assumed corresponding mostly to the viremic period (1/*γ*), where the long term effects of sexual infectiousness in males are not likely to be relevant.

As the Reproductive Number (R) is defined by (4), we used the analogous Eqs (5) and (6) to represent the dynamics between the classes.

$$R=\frac{\beta }{\gamma }$$ (4)

$$\frac{dS}{dt}=\frac{-\gamma RIS}{N}$$ (5)

$$\frac{dI}{dt}=\frac{\gamma RIS}{N}-\gamma I$$ (6)

Because we had the opportunity to combine the evidence on Zika incidence in women in the reproductive age bracket with the number of lost births associated with Zika from the birth records, we based the inference on the female epidemic (restricted to fertile age) and then derived the parameter for the total female and male epidemic from that of females in fertile age estimates.

It is important to mention that during the inference we did not simulate the dynamical model, but rather use its contraints in the bayesian inference model as detailed below.

In this paper we used the term observability most of the time, as we wanted to make a clear distinction between notified cases (necessarily observed) and those not notified because patients never sought medical attention, or were not recognized as a Zika case. We assumed the latter group to consist mostly of asymptomatic and very mild Zika cases, given the expected high ratio of asymptomatic to symptomatic cases [4, 10].

### The data set

The data used in this paper comes from two public health information systems, *Sistema de Informção de Agravos e Notificação* (SINAN) and *Sistema de Informações sobre Nascidos Vivos* (SINASC).

From SINAN we extracted the Zika incidence ranging from the first epidemiological week of 2015 to the end of 2016. The registry for live births, SINASC, is a very well kept and complete registry, from which we extracted the total number live births recorded from 2012 to 2016. Both sets of data used had been fully anonymized prior to access by the authors.

In our study, we used official population estimates, calculated from the last census in 2010 for men (*N*_*m*_ = 2, 994, 018) and women (*N*_*f*_ = 3, 392, 425).

#### Data preparation

The incidence data extracted from the SINAN were aggregated weekly and separated by sex ($D_{f}$ and $D_{m}$) and the subset of reports in fertile age ($D_{ffa}$—between 14 and 48 years old) was selected from the female data ($D_{f}$).

Splitting the incidence data in this way was important because we exploit the relation between the decrease in number of births (in the studied period) and the sexual transmission of Zika virus. In addition, we already know that the sexual transmission of the Zika virus is stronger from male to female [11]. That way, the $D_{ffa}$ dataset is the cornerstone on which we built the mathematical model for inferring the other fractions of populations, represented by their respective data stratus.

From the SINASC data, we define $D_{mz}$. This time series is used as proxy for the weekly number of lost births due to Zika in Rio de Janeiro in 2016. Arboviroses transmitted by Aedes aegypti display marked seasonality, with the incidence peaking at the end of summer.

We hlrestricted the inference to the data within the epidemic season. In order to delimit the epidemic season, we assume the incidence data is a poison process (Eq (7)) with two distinct intensities, a lower intensity corresponding to off-season and a higher average intensity corresponding to the epidemic season.

$$O_{t}∼Poisson\left(\lambda_{t}\right)$$ (7)

The Poisson process intensity λ can be estimated from the data. We make the simplifying assumption that λ_*t*_ is constant in the high and low seasons, we have to estimate three values for λ (Eq (8)) and two additional parameters *τ*_1_ and *τ*_2_ corresponding to the transition times as showed in the random variable *O* (Eq (9)), where $1_{\left(condition\right)}$ is the indicator function.

$$\lambda =\{\begin{array}{cc} \lambda_{1} & \;\text{if}\;t\leq \tau_{1}\\ \lambda_{2} & \;\text{if}\;\tau_{1}\leq t\leq \tau_{2}\\ \lambda_{3} & \;\text{if}\;\tau_{2}\leq t \end{array}$$ (8)

$$O|\lambda ,\tau ∼Poisson(\lambda =1_{\left(week\leq \tau \right)}\cdot \lambda_{i}+1_{\left(week>\tau \right)}\cdot \lambda_{j})$$ (9)

**Fig 1:**
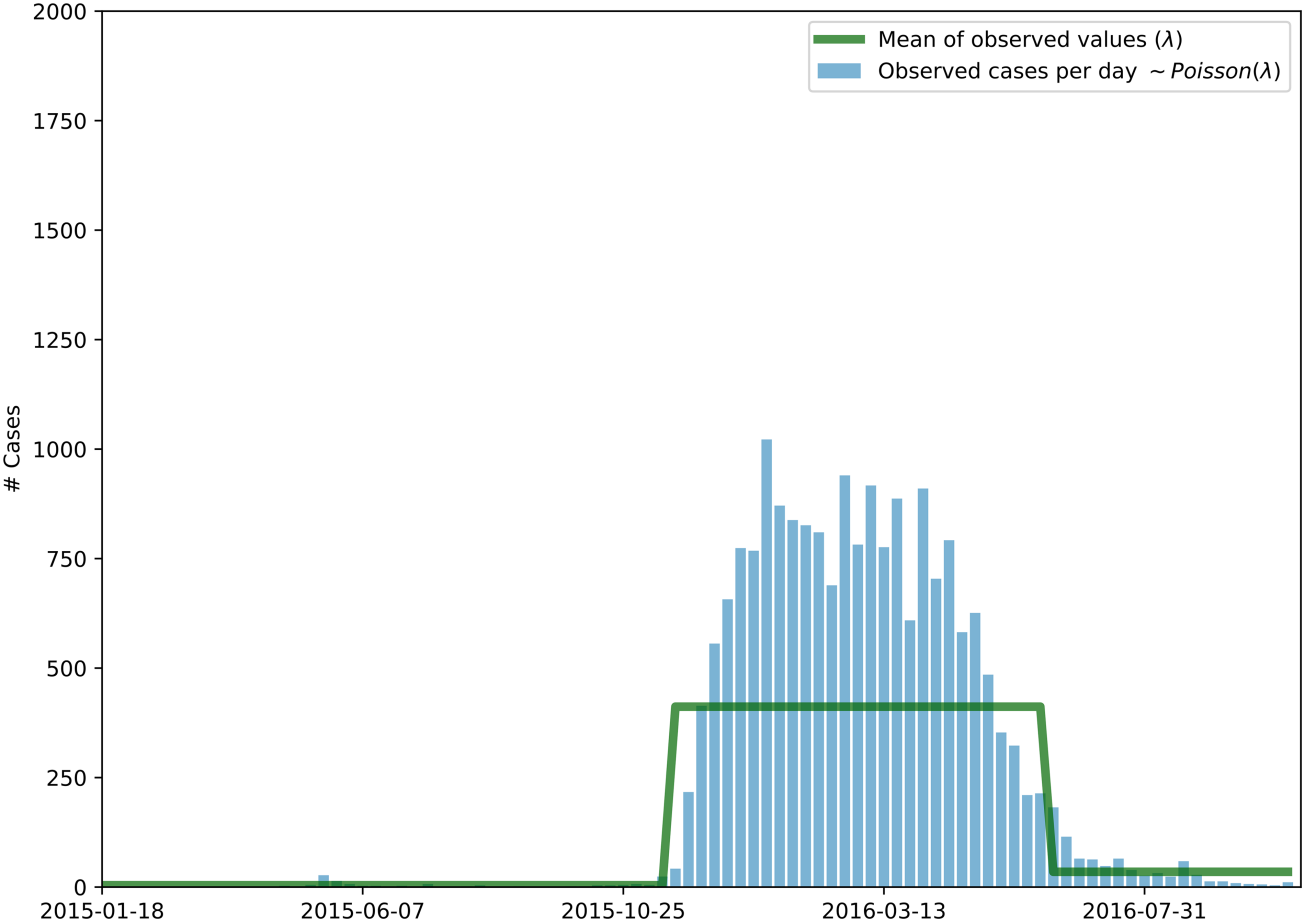
Period of interest. Given that the Zika’s reports follow Poisson distribution, the green curve is the value of the parameter of the distribution in each week, the points of maximum in the interval bounds the period of significant infection, being thus the period that will be analyzed.

**Fig 2:**
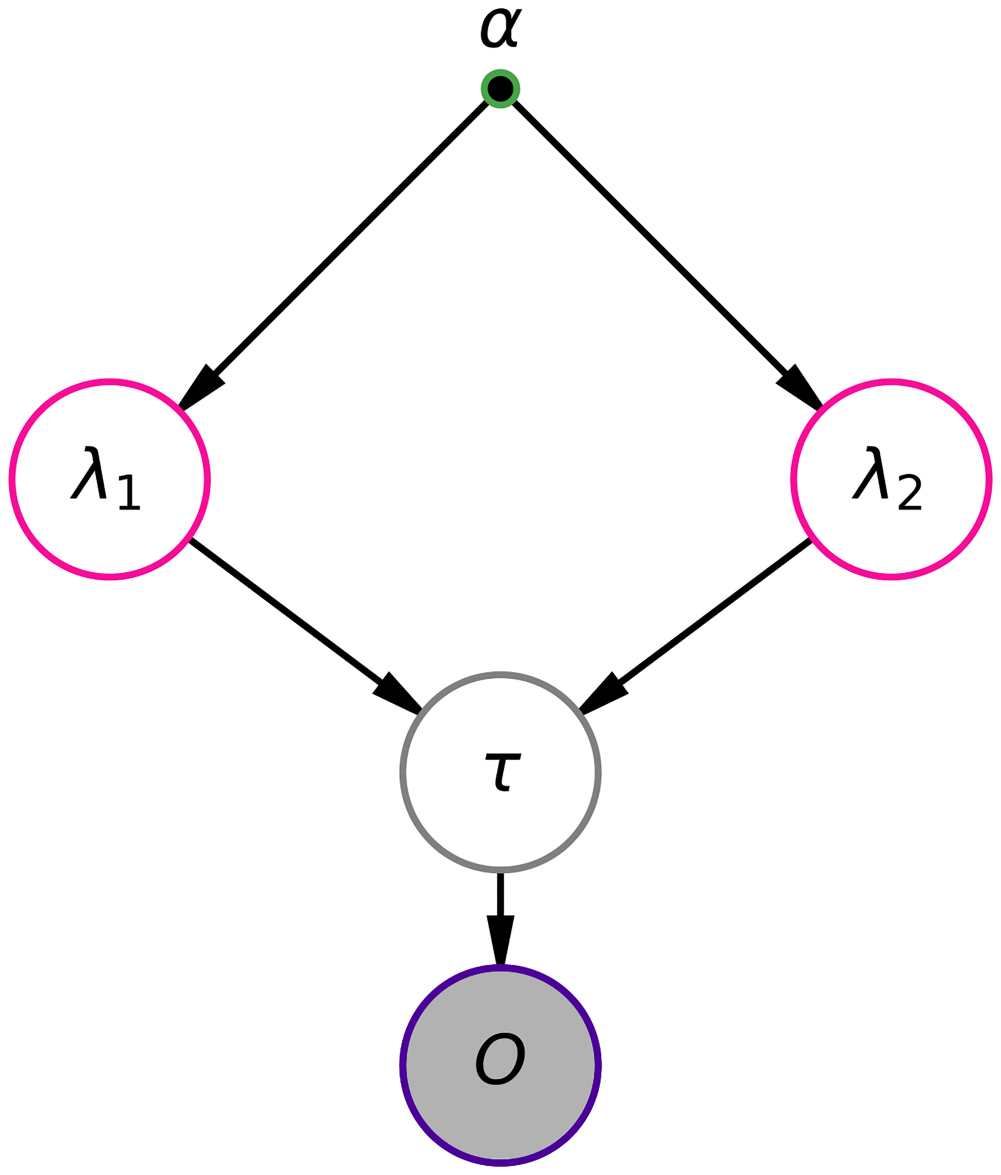
Bayesian network for estimating the time boundaries of the epidemic season. The gray filled circle means the observed data, the green border dots to represent a fixed parameter, the gray border circle indicate a *Uniformly* distributed variable, the pink border variable indicate *Exponentially* distributed variables and the purple halo means *Poisson* distribution. The arrows indicate probabilistic dependencies.

That way, the epidemic season becomes bounded by the transition points *τ*_1_ and *τ*_2_ as shown in Fig 1. The inference problem can be depicted by the probabilistic graphical model of Fig 2. We applied a MCMC-based Bayesian inference procedure to this model to estimate its latent variables.

The prior distribution of *τ* (Eq (10)) is modeled as a discrete uniform on the interval $\left[0,\hat{n}\right]$, where $\hat{n}$ is the number of weeks in $D_{1}$.

Since λ_*k*_ (*k* ∈ {1, 2, 3}) is the mean of a Poisson random variable, it must be positive definite. Thus, we choose exponential distributions to the priors, as shown in Eq (11). Also, the hyper-parameter in each λ_*k*_ was arbitrarily chosen to be equal to the inverse of the average of the count data (a usual procedure for exponential parameters).

The prior distributions of the latent variables are given below.

$$\tau ∼U\{0,\hat{n}\}$$ (10)

$$\lambda_{k}∼Exp\left(1/\bar{y}\right)$$ (11)

The estimated bounds of the epidemic season, *τ*_1_ and *τ*_2_, were used to temporally clip $D_{1}$. By retaining only case reports for females in fertile age, we obtained $D_{ffa}$. After this we were left with *n* = 29 weeks of data to analyze.

The same time window was applied to the remaining case sets $D_{f}$, $D_{m}$ and D, respectively the total female cases, male cases and the total population cases.

We followed the procedure described in [2] to generate a weekly count of missing births or miscarriages ($D_{mz}$).

Simply put, the procedure consists in subtracting the number of births in the i-th week of 2016 from the i-th week of 2015, as shown by the Eq (12), where *B*_*j*_ is the array of weekly number of live births in the *j*-th year.

$$D_{mz}^{\prime }=B_{2015}-B_{2016}$$ (12)

**Fig 3:**
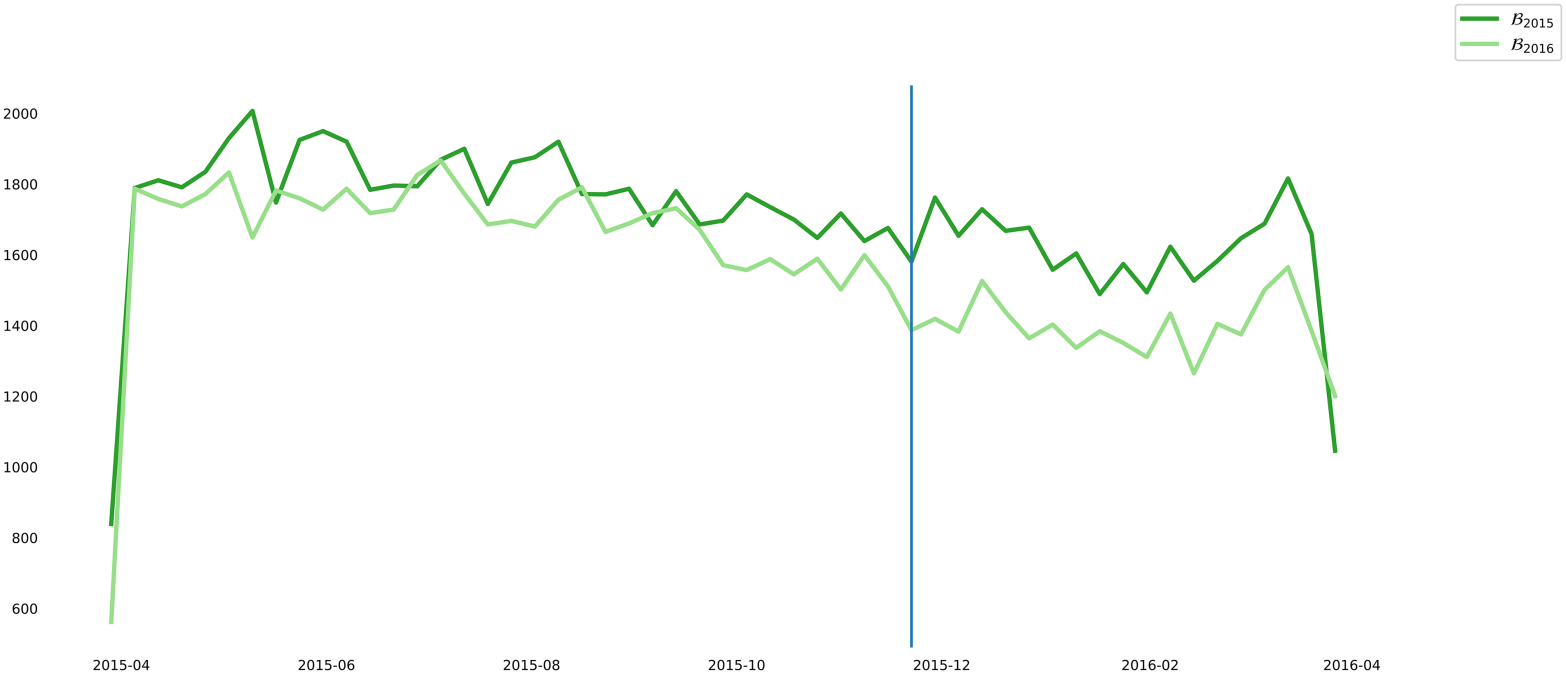
Overlapping of time series with births in 2015 and 2016. The vertical line marks the 40-th week.

According to [2] the loss of births trails the Zika epidemic by about 40 weeks, as pictured in the Fig 3. Thus, $D_{mz}^{\prime }$ was shifted 40 weeks backward and bounded to the same time window as $D_{ffa}$. The weeks with no data in the bounded $D_{mz}^{\prime }$ were filled with 0 (assuming no losses), thus generating $D_{mz}$.

### Likelihoods

The case report data in each population fraction was modeled as a Poisson random variable Δ*S*_*o*_ (Eq (13)), whose parameter is the product of the incidence (Δ*S*) and the probability of observation (*po*).

Let *pp* be the probability of a woman (of fertile age) being pregnant and *mz* the probability of one of these woman losing her baby due to Zika. Therefore, $D_{mz}$ was modeled as a Poisson random variable (M) with parameter Δ*S* ⋅ *pp* ⋅ *mz*, as shown in Eq (14). This likelihood was used only in the female in fertile age model.

$$\Delta S_{o}∼Poisson\left(\Delta S\cdot po\right)$$ (13)

M∼Poisson(ΔS·pp·mz) (14)

### Female in fertile age model

The first model we ran was just for female population in fertile age. The following priors where used.

We know from [7] that 11.5% (95%CI: 7.32–17.9%) of infections were reported overall in that study. Thus, assuming that the mildness of most Zika cases could lead to similar levels of underreporting in our data, we defined the prior probability of observation (*po*) to follow a beta distribution as per Eq (15), where the parameters *α*_*po*_ and *β*_*po*_ were chosen so that the mean and the standard deviation agree with the mean and 95% credible interval previously reported.

As established in [2], there is a strong association between the reduction in the observed fertility in the period of analysis (2016) and the Zika epidemic in the same year. With this knowledge, we assumed that the percentage of fertility reduction caused by Zika (*mz*) follows a beta distribution whose parameters *α*_*mz*_, and *β*_*mz*_ imply a mean of 0.5 and 95% probability between 0.25 and 0.75. The distribution of *mz* is given by Eq (17).

The variable *pp* (Eq (16)) represents the probability of a woman in fertile age being pregnant. We choose a beta prior with mean equal to the ratio between the number of births in 2015 and the number of women in fertile age.

For the SIR model state variables, we proposed vector shaped priors since we fitted the entire series to data at once. We chose prior probabilities functions that fit the definition of each variable.

The parameter $R_{t}$ (Eq 19), which represents the ratio between the transmission rate of an individual and the period in which it remains infectious, was modeled by a vector-shaped random variable, with each element of the vector representing one week of the studied period. We allowed for its value to change from week to week to accommodate the effect of fluctuating environmental conditions not explicitly included in the model. Also, we used as prior a gamma distribution such that the parameters *α*_*r*_ and *β*_*r*_ follows the results of [7] in the the Moorea region (mean 4.8 and (95%CI: 3.2–8.4)).

We assumed that the infectivity period (*γ*^−1^) was constant throughout the year and follows a gamma distribution with an average of 7 days and a standard deviation of 3.5. This choice was made to cover the infectious period and the life span of the mosquito (5 and 14 days respectively, as shown in [7]). This prior is summarized by the variable (18), the parameters *α*_*γ*_ and *β*_*γ*_ follow the restrictions given above.

$$po∼Beta(\alpha_{po},\beta_{po})$$ (15)

$$pp∼Beta(\alpha_{pp},\beta_{pp})$$ (16)

$$mz∼Beta(\alpha_{mz},\beta_{mz})$$ (17)

$$\gamma^{-1}∼Gamma\left(\alpha_{\gamma },\beta_{\gamma }\right)$$ (18)

$$R_{t}∼Gamma\left(\alpha_{r},\beta_{r}\right)$$ (19)

With no prior information about the weekly total number of infected persons (*I*), we chose a more flexible and decentralized model to this variable, capable of, using a Hamiltonian-based sampler as No-U-Turn Sampler (NUTS) (described in [12]), to efficiently span the sample space to gather points of higher probability [13].

Assuming that the number of infected individuals follows a well behaved concave function, we fitted a quadratic polynomial that represents this population at each week, according to Eq (29).

Thus, our prior for the compartment *I* (29) was governed by a second degree polynomial whose coefficients *a* (23) and *b* (27) are decentralized variables defined by normally distributed central values with mean 0 and standard deviation 10000 *a*_*μ*_ (20) and *b*_*μ*_ (24), plus a perturbation characterized by *a*_*o*_ ⋅ *a*_*σ*_ and *b*_*o*_ ⋅ *b*_*σ*_ respectively and a coefficient *c* (28) normally distributed with mean equal to the *pim* ⋅ *N* and standard deviation 10000.

The constant (*pim* = 58/16191) is the percentage of infected at time (t = 0), calculated as the ratio between the number of infected at time (t = 0) on Moorea Island [7] and the total population of Moorea Island.

The offsets *a*_*o*_ (22) and *b*_*o*_ (26) are multidimensional standard normally distributed variables that scale the random variables *a*_*σ*_ (21) and *b*_*σ*_ (25), Half-Cauchy distributed variables that represents the standard deviation of *a*_*μ*_ and *b*_*μ*_ respectively. The Half Cauchy is a typical choice of prior for standard deviation variables to keep the inference sensitive to weakly-informative prior distribution [14].

Therefore the size of the population in the infectious class on the *t*-th week was summarized in the quadratic function (29), a decentralized procedure to make easier to the MCMC algorithm scan areas in the space with high curvature, as described by [13].

$$a_{\mu }=N\left(0,10000\right)$$ (20)

$$a_{\sigma }=Cauchy\left(0,5\right)\;\left(a_{\sigma }\geq 0\right)$$ (21)

$$a_{o}^{t}=N\left(0,1\right)$$ (22)

$$a=a_{\mu }+a_{o}*a_{\sigma }$$ (23)

$$b_{\mu }=N\left(0,10000\right)$$ (24)

$$b_{\sigma }=Cauchy\left(0,5\right)\;\left(b_{\sigma }\geq 0\right)$$ (25)

$$b_{o}^{t}=N\left(0,1\right)$$ (26)

$$b=b_{\mu }+b_{o}*b_{\sigma }$$ (27)

c=N(pmi·N,1000) (28)

$$I_{t}=a_{t}\cdot t^{2}+b_{t}\cdot t+c$$ (29)

As the time interval (Δ*t*_*i*_) between points in our time series was constant and equal to a week, we can set (30) equal to 1.0 and carry out our study using finite differences.

$$\Delta t_{i}=1,\;\left(0\leq i\leq n\right)$$ (30)

The approximation (31), from the equalities (3) and (30), made it evident that the *t*-th inflow to the *R* compartment is computed as the population in the *I* compartment on the *t*-th week divided by *γ*^−1^.

$$\Delta R_{w}\approx \left(\gamma \cdot I_{t}\right)=\frac{I_{t}}{\gamma^{-1}}$$ (31)

Also, we know that once in the *R*-compartment, there is no outflow. So the population in the *w*-th week (*R*_*w*_) is the population in the (*w* − 1)-th week plus the input stream (*R*_*w*_ = *R*_*w*−1_ + Δ*R*_*w*_). In other words, *R* is the cumulative sum of the input stream in each period (week), given by the Eq (32) (assuming *R*_0_ = 0).

Considering that the population in Rio de Janeiro (*N*) was assumed constant, the population *S* in the *w*-th week (*S*_*w*_) was determined by the Eq (33).

Therefore, the incidence function in the study interval was given deterministically by Eq (34).

$$R_{w}=\sum_{i=1}^{w}\Delta R_{i}$$ (32)

$$S_{w}=\left(N-R_{w}-I_{w}\right)$$ (33)

$$\Delta S^{w}=\frac{R_{t}\cdot S_{w}\cdot I_{w}}{\gamma^{-1}\cdot N}$$ (34)

The Table (1) summarizes the prior distributions used for females in fertile age model.

**Table 1:** Prior distributions of the parameters used in the model.

*Parameter*	*Definition*	*Prior*	*References*
*po*	Probability of report (observance)	Beta(17.4, 134.2)	[7]
*mz*	Percentage of fertility decline caused by Zika	Beta(12.0, 12.0)	[2]
*γ*^−1^	Infectious Period	Gamma(14.0, 2.0)	[7]
$R_{t}$	Reproductive Number	Gamma(15.4, 3.2)	[7]
*pp*	Probability of pregnancy	Beta(100.1, 1579.7)	
*a*_*μ*_, *b*_*μ*_	Mean of the parameters *a* and *b*	N(0,10000)	
*a*_*σ*_, *b*_*σ*_	Standard deviation of the parameters *a* and *b*	*Cauchy*(0, 5)	
$a_{o}^{t},b_{o}^{t}$	Offset of the parameters *a* and *b*	N(0,1)	
*c*	Intercept of the Infectious compartment	N(pmi·N,10000)	

For the Male, Female and Total models, we have set the parameter values related to pregnancy (*pp* and *mz*) to the posterior means obtained in the Female in Fertile Age model.

## Results

### Epidemiological parameter estimates

**Fig 4:**
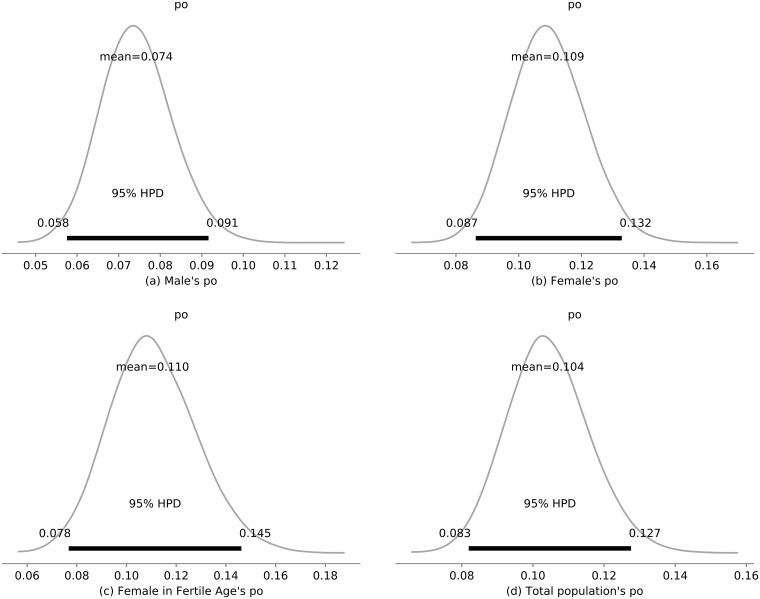
Probability of observation. Posterior probability distribution of the probability of observation in each sub-population model. In particular, the mean and highest 95% posterior density interval of each parameter.

The Fig 4 shows the posterior distributions of the probability of observing (reporting) a case of zika for each of the subgroups (Male—(a), Female—(b), Female in Fertile Age—(c) and Total Population—(d)) during 2016 in Rio de Janeiro. The probability of observation for men was noticeably lower than for women.

**Fig 5:**
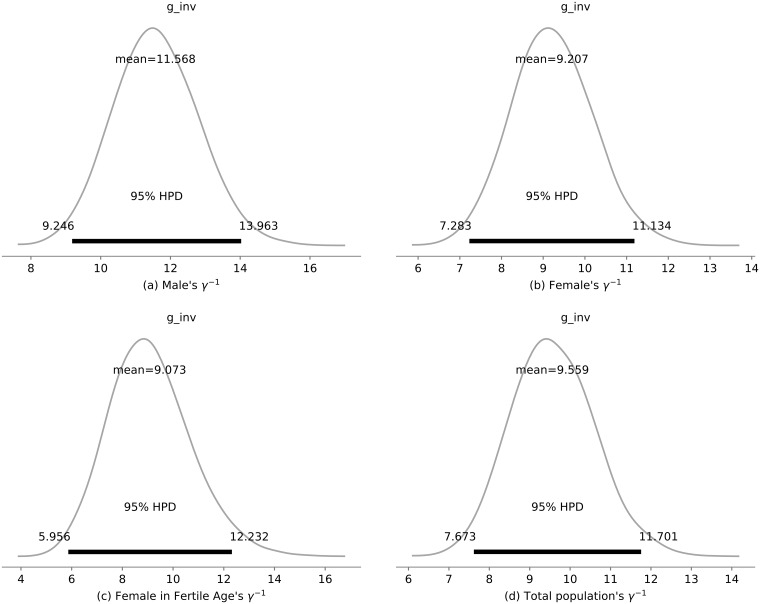
Infective period (*γ*^−1^). Posterior probability distribution of the *γ*^−1^ in each sub-population model. In particular, the mean and highest 95% posterior density interval of each parameter.

Fig 5 summarizes the posterior distributions of the infectious period for each of the subgroups (Male—(a), Female—(b), Female in Fertile Age—(c) and Total Population—(d)). As expected, the results indicated that, on average, the male and female infectious periods were the same. Also, the results indicated a small displacement to the right of what was established a priori, due to the mosquito life span and the infectious period by sexual transmission (implicitly attached in our model to the vector transmission process).

**Fig 6:**
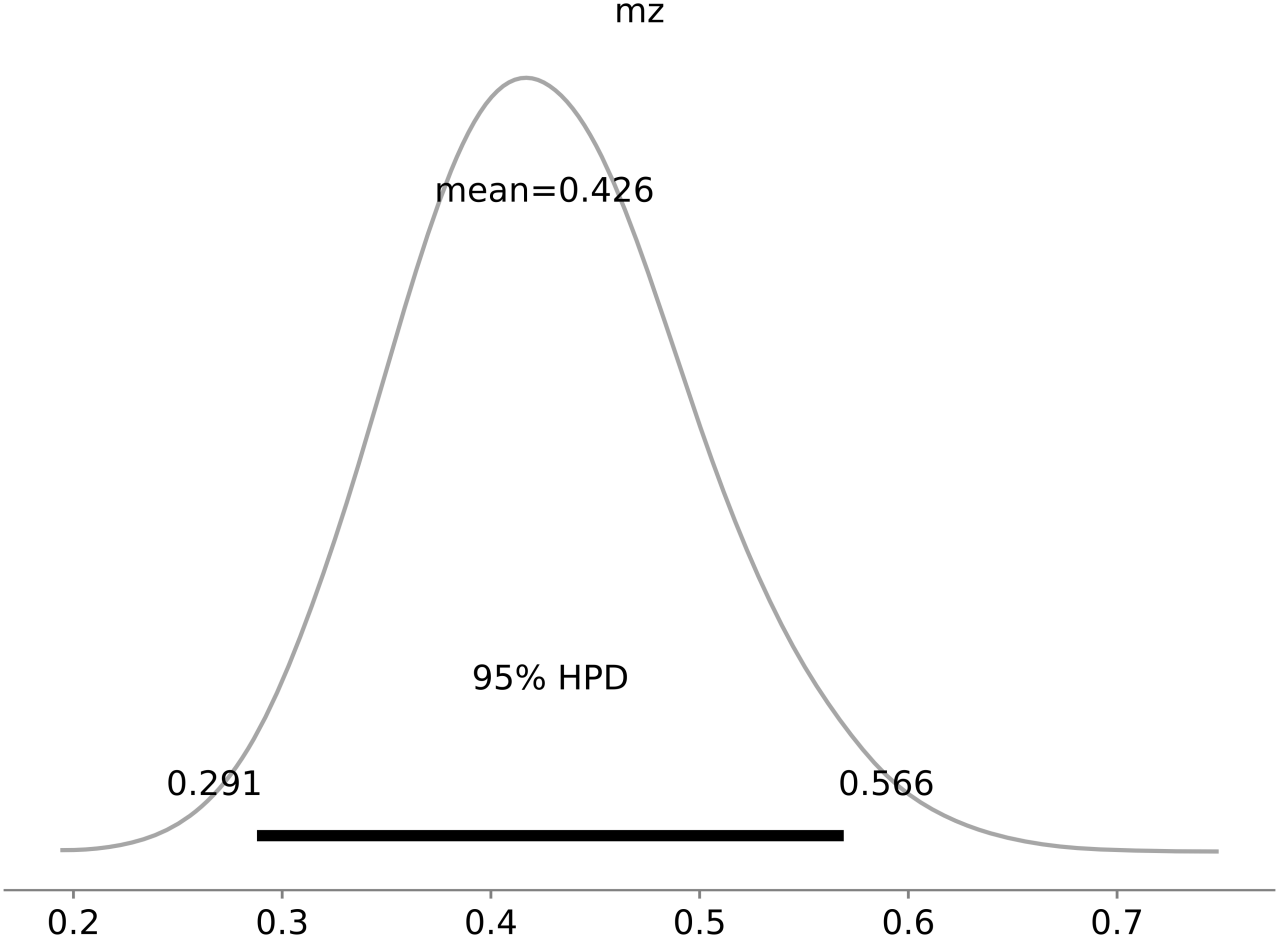
Estimated value of the quota on fertility reduction in 2016 due by Zika (*mz*).

Our results indicated that Zika had a 42.6% (95%CI: 29.1–56.6%) participation in reduction of fertility in 2016 as shown in Fig 6.

**Fig 7:**
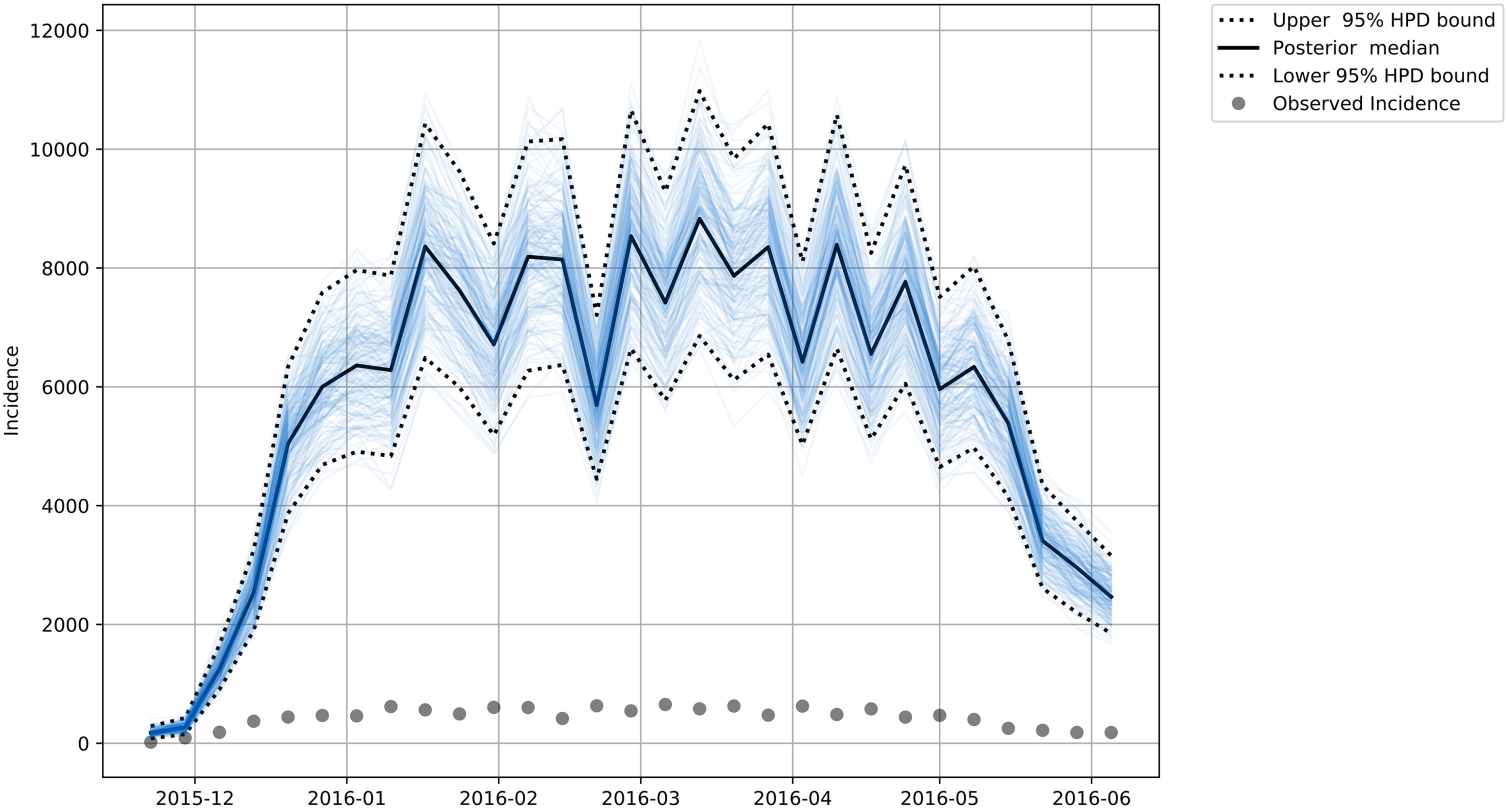
Estimated Total Zika male incidence. The dashed lines bounds the 95% credibility range and the continuous darker curve represents the median of the total male incidence. The dots are the observed incidences in our data.

**Fig 8:**
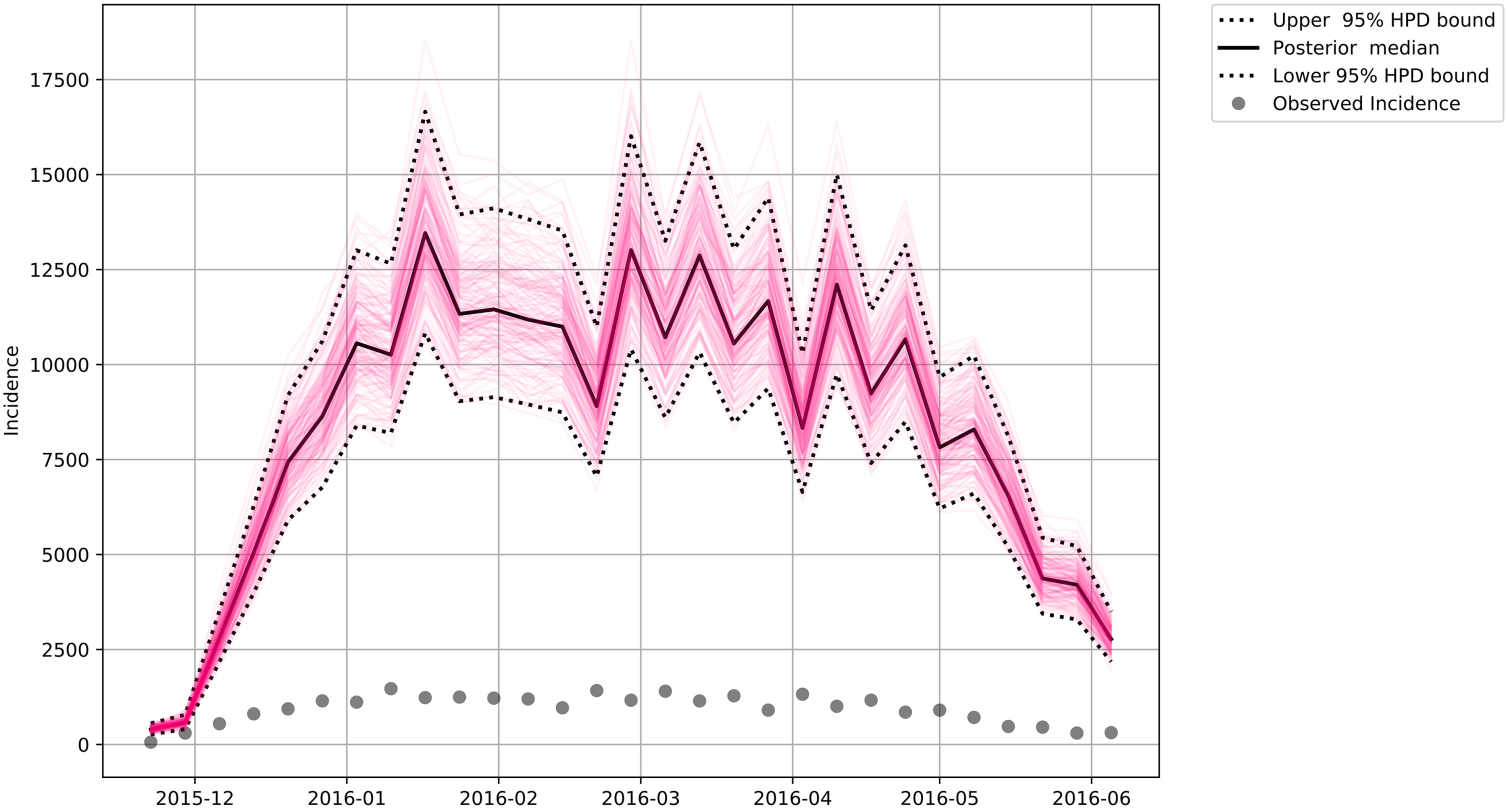
Estimated Total Zika female incidence. The dashed lines bounds the 95% credibility range and the continuous darker curve represents the median of the total female incidence. The dots are the observed incidences in our data.

**Fig 9:**
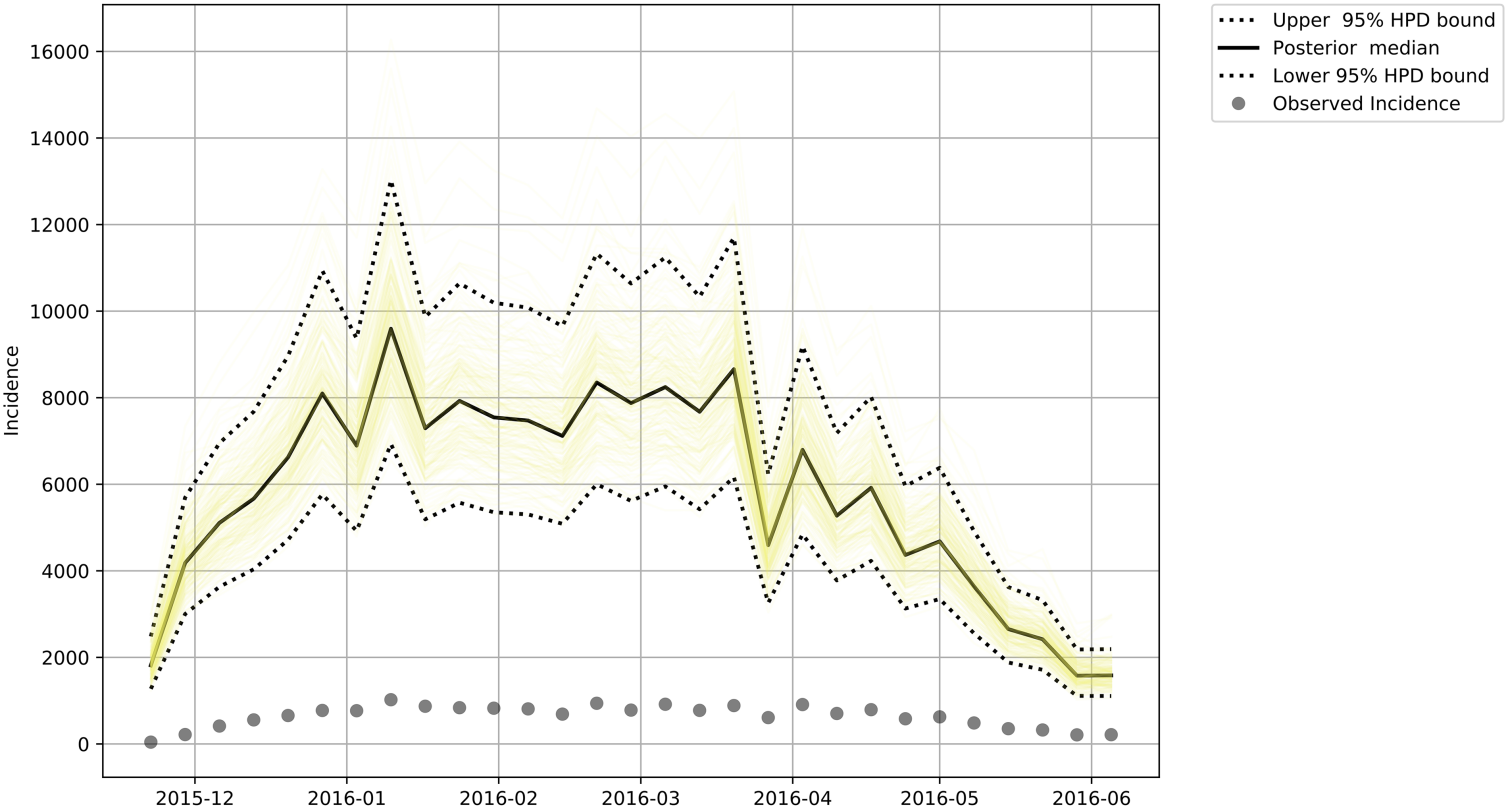
Estimated Total Zika Female in Fertile Age Incidence. The dashed lines bounds the 95% credibility range and the continuous darker curve represents the median of the total female in fertile age incidence. The dots are the observed incidences in our data.

**Fig 10:**
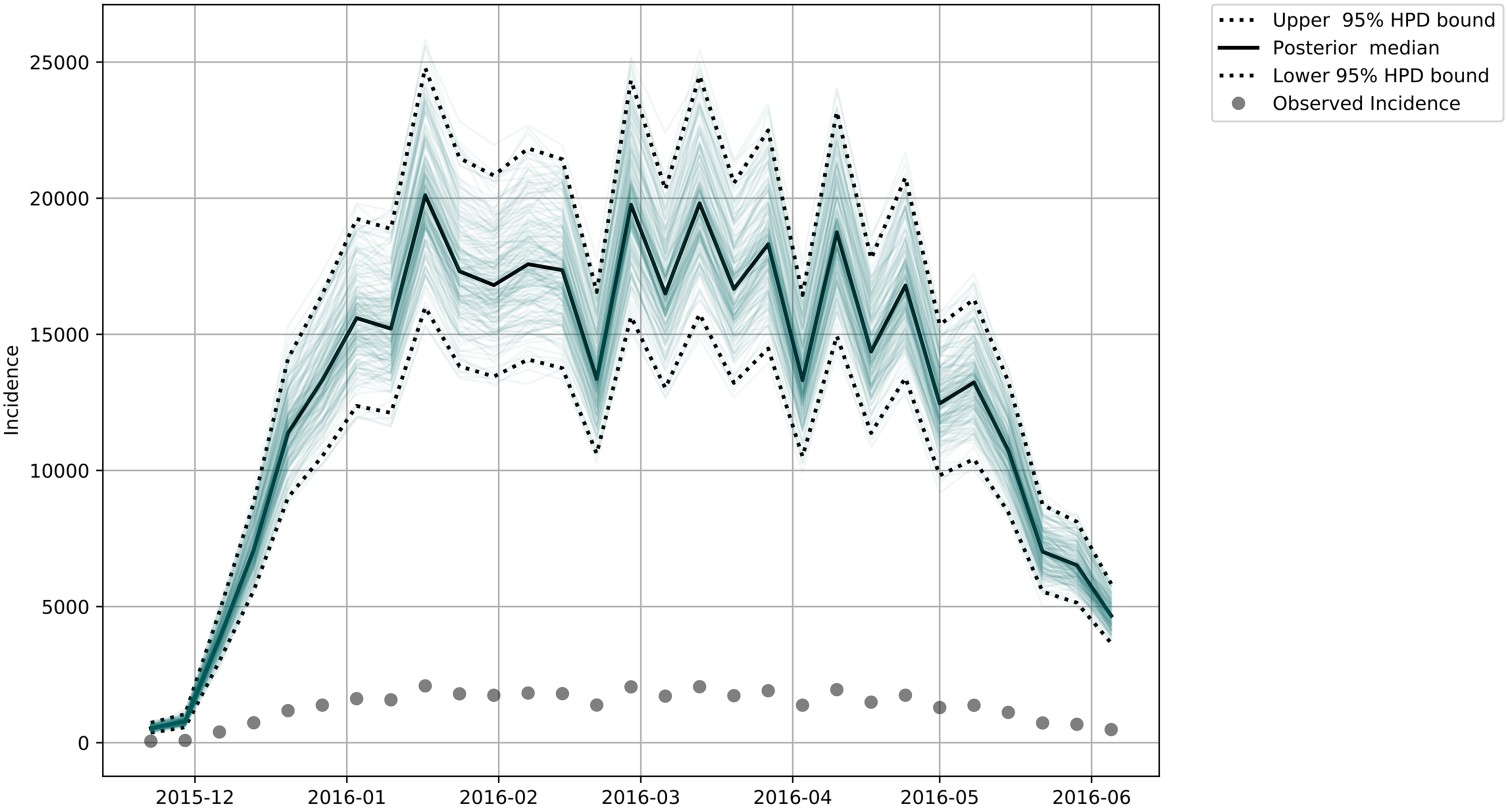
Estimated Total Incidence. The dashed lines bounds the 95% credibility range and the continuous darker curve represents the median of the total incidence. The dots are the observed incidences in our data.

We estimated the total incidence for each of the subgroups (Male—Fig 7, Female—Fig 8, Female in Fertile Age—Fig 9 and Total Population—Fig 10) reported and unreported in the analyzed period.

**Fig 11:**
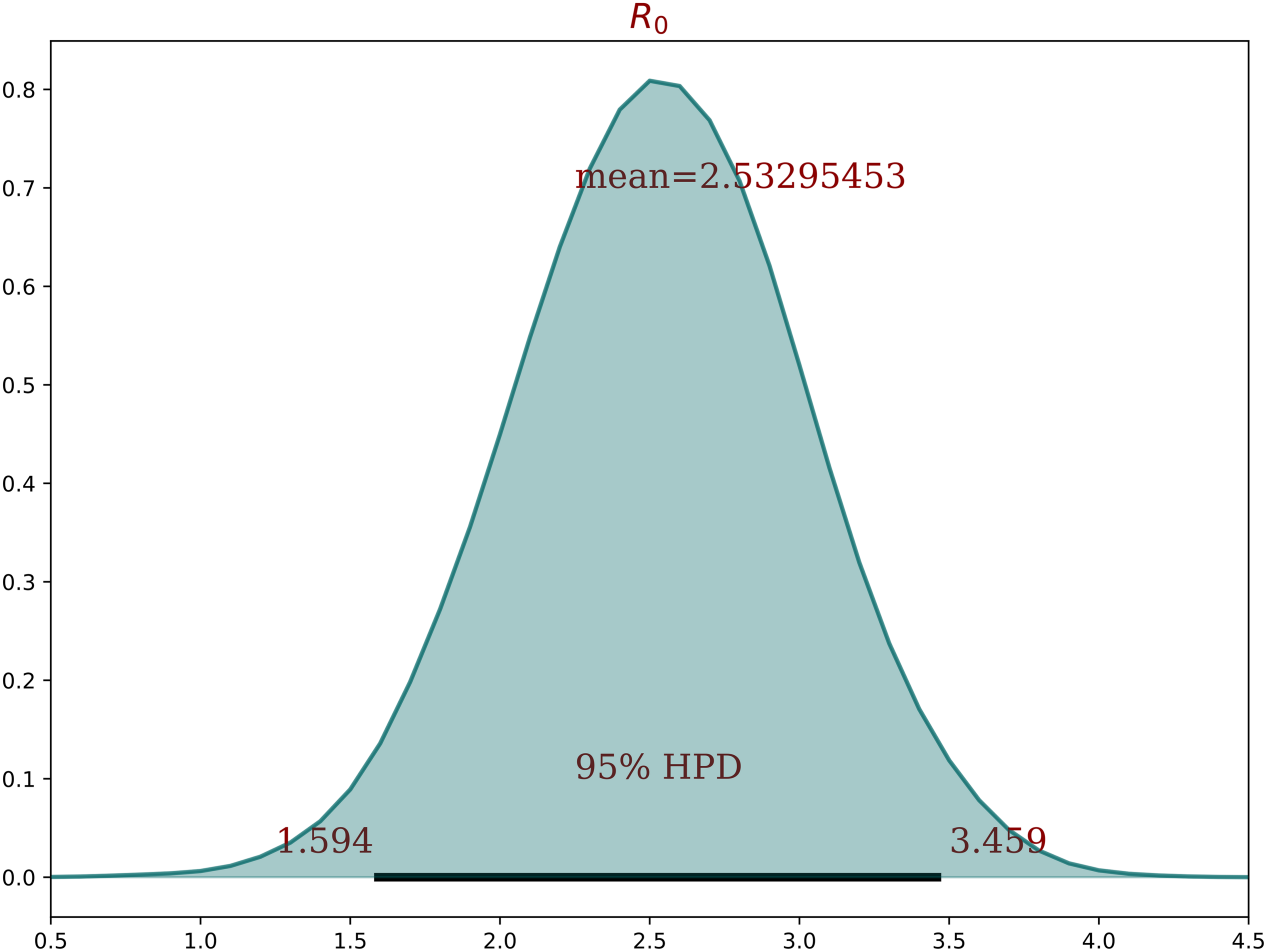
Basic reproductive number ($R_{0}$). Posterior distribution of the basic reproductive number of the Zika epidemic in Rio de Janeiro in 2016.

On Fig 11 we can see the distribution of the basic reproductive number $R_{0}$, for the Zika epidemic in total population of Rio de Janeiro city in 2016.

## Discussion and conclusion

In this study, a new approach was developed for inferring the parameters that govern the spread of the Zika virus within Rio de Janeiro in 2016 and for estimating its real reach.

In our study, we found that only 10.4% (95% CI: 8.3–12.7%) of the total infections were reported. This result agrees with the estimated fraction of the population reported in other regions [7]. Also we verified that from the drop in the birth rate in 2016 compared to 2015, 42.6% (95% CI: 29.1–56.6%) was caused by Zika virus, and a really significant contribution caused by reasons like miscarriage and avoided pregnancies.

Our results show that a person remains infectious for 9.599 (95% CI: 7.673–11.701) days, which agrees with previous studies [7, 15], despite the fact that we did not include vector transmission in the model.

Knowing the full size of an epidemic is very relevant to understanding the full impact of a disease both in terms of morbidity and also in consequent economic losses. Particularly for Zika, the asymmetry in its dissemination caused by sexual transmission from men to women of fertile age poses an additional challenge since we cannot assume the same transmissibilities for both sexes. Different perceptions of risk for men and women lead to different underreporting rates by gender for the epidemic as a whole. The impact of Zika on the early miscarriage rate turned the live-birth record into a key source of information allowing us to estimate the underreporting rates. The fact that the birth record is complete made it possible for us to properly measure the epidemic.

Several favorable factors made it possible for us to study this phenomenon in Rio de Janeiro: detailed data at the level of individual cases, a large epidemic in a fully susceptible population and very stable fertility rates due to the large population.

The use of a SIR transmission model as a constraint for the inference on the fully realized dynamics without requiring the simulation of the entire model dynamics at each MCMC step, is an original approach, which greatly reduces the computational cost of the entire inferential process. We believe that using mechanistic transmission models as a basis for inferring latent parameters in the context of communicable disease dynamics, is a key to ensuring a causal interpretation of the results because these causal mechanisms are explicitly stated.

Although estimating $R_{0}$ was not the main focus of this study, the values obtained (2.53% (95% CI: 1.59−3.45%)) are consistent with previous estimates [8, 16, 17].

Thus, although we applied a simpler epidemiological model, the statistical model was efficient enough to obtain results similar to those obtained in other studies with more detailed epidemiological models.

Knowing the full size of the Zika epidemic in Rio in 2016, is very important if we are to fully understand the risks of having a second wave of Zika in the city in the near future. In 2017, there was no outbreak of Zika in Rio, despite the continued low-intensity transmission [18].

Although our model presents general results similar to those of other studies, it does not provide interpretations of the mosquito’s role in the vector transmission process, nor does it give the magnitude of the sexual contribution in the transmissive process. These are limitations of our work and on the understanding of the of virus spreading dynamics.

In this paper we sought to provide answers to the very important issue of the true size of he epidemic. The next natural step would be to use the same dataset to generate estimates for the parameters of a full vector and sexual transmission model.
